# Functional and anatomical outcomes of Yamane technique-based scleral fixation after complicated cataract surgery versus IOL extraction

**DOI:** 10.1186/s12886-026-04876-9

**Published:** 2026-06-02

**Authors:** Fatih Kahramanoğlu, Muzaffer Talha Albayrak, Serdal Ayas

**Affiliations:** 1 Department of Ophthalmology, Adana City Training and Research Hospital, Adana, Türkiye; 2 Department of Ophthalmology, Kayseri State Hospital, Kayseri, Türkiye

**Keywords:** Yamane technique, Scleral fixation, Secondary intraocular lens implantation, Complicated cataract surgery, Intraocular lens extraction, Aphakia

## Abstract

**Purpose:**

To evaluate and compare the anatomical and functional outcomes of Yamane scleral-fixated intraocular lens (IOL) implantation in aphakic eyes following complicated cataract surgery (CCS) or prior IOL extraction (IE), using standardized outcome measures.

**Methods:**

This retrospective study included 50 eyes of 50 patients who underwent Yamane scleral-fixated IOL implantation (CCS: *n* = 13; IE: *n* = 37). Best-corrected visual acuity (BCVA), intraocular pressure (IOP), refractive outcomes, endothelial cell density (ECD), and central macular thickness (CMT) were evaluated preoperatively and at 6 months postoperatively. Standardized changes (ΔlogMAR, ΔECD%, ΔCMT%) were calculated.

**Results:**

BCVA and spherical equivalent improved significantly in both groups (*p* < 0.001), with no significant difference in visual gain between groups (*p* = 0.063). Absolute astigmatism decreased significantly in the IE group (*p* = 0.036) but not in the CCS group (*p* = 0.124). IOL-related astigmatism remained low and comparable between groups (*p* = 0.765). IOP decreased significantly in the CCS group (*p* = 0.036) but remained stable in the IE group (*p* = 0.476). ECD decreased significantly in both groups, with no between-group difference in percentage loss (*p* = 0.382). CMT remained stable, with no significant differences (*p* = 0.974). Complication rates were low, with no severe adverse events.

**Conclusion:**

Yamane scleral fixation provides safe and effective short-term anatomical and functional outcomes in aphakic eyes following both CCS and IE. Despite differences in surgical history, outcomes were largely comparable between groups across most parameters. Intraocular pressure decreased in eyes undergoing CCS and remained stable in previously operated eyes, reflecting expected physiological responses. These findings further support the Yamane technique as a reliable and versatile option for secondary IOL implantation, with outcomes largely independent of prior lens status.

**Clinical trial number:**

This study is a retrospective study conducted using data obtained from patient records. It does not involve an interventional clinical trial; therefore, a clinical trial registration number is not applicable.

**Supplementary Information:**

The online version contains supplementary material available at 10.1186/s12886-026-04876-9.

## Introduction

The zonular fiber network connects to the ciliary body and the lens equator, forming a 360° ring. Zonular fibers are the most important anatomical structure that stabilizes the lens in phakic eyes and the intraocular lens (IOL) in pseudophakic eyes [1, 2]. Loss or weakness of zonular fibers can lead to lens subluxation in phakic eyes and IOL subluxation in pseudophakic eyes [3, 4]. While lens subluxation can result from various etiologies, including trauma, inherited disorders such as Marfan syndrome, or systemic diseases, IOL subluxation may arise from trauma, zonular fiber damage during previous cataract surgery, severe capsular fibrosis, pseudoexfoliation, uveitis, high myopia, or other conditions associated with progressive zonular weakening and capsular contraction [3, 5, 6]. In these cases, patients often experience progressive blurred vision, monocular diplopia, or secondary angle-closure glaucoma [7]. Aphakia following the extraction of a subluxated lens or IOL represents a significant surgical challenge [8–10]. For the management of aphakia, since the introduction of flanged intrascleral fixation by Yamane et al., various modifications have been developed, each aiming to improve specific aspects of the original technique [11–14]. This study aims to assess the anatomical and functional outcomes of secondary intraocular lens (IOL) implantation using the Yamane technique in eyes rendered aphakic following either complicated cataract surgery (CCS) or prior IOL extraction (IE). To our knowledge, this study is one of the few to directly compare outcomes of Yamane scleral fixation between eyes undergoing the procedure after complicated cataract surgery and those undergoing it following IE. In addition, the present study provides a standardized comparison by evaluating percentage endothelial cell loss and refractive outcomes under similar surgical conditions, offering clinically relevant insights into the impact of prior surgical status on postoperative outcomes.

## Methods

This retrospective study included 50 eyes from 50 patients who underwent Yamane IOL implantation due to IOL subluxation or lens subluxation between January 2023 and June 2025 at Adana City Training and Research Hospital. Patients were divided into two groups: those who had undergone complicated cataract surgery (CCS) or prior IOL extraction (IE). All surgical procedures were performed by a single surgeon (F.K.). In all cases, a three-piece intraocular lens (Eyecryl, manufactured by Biotech Vision Care) was used. All surgeries were performed under sub-Tenon anesthesia. In all cases, the Yamane technique was applied with transconjunctival sclerotomies created 2 mm posterior to the limbus at the 3 and 9 o’clock positions for symmetric haptic externalization. The 27-gauge needle was introduced at an approximately 20–30° angle relative to the scleral surface to facilitate controlled haptic externalization. The detailed surgical technique is described below. The presence of pseudoexfoliation (PEX), an important predisposing factor, as well as a history of ocular trauma, were recorded from patient files. All patients were routinely examined at postoperative day 1, week 1, month 1, and month 6. In order to present the short-term outcomes of the study, the 6-month results were reported.

Keratometric and spherical/cylindrical refractive values were obtained in all cases using an autorefractometer (VISUREF 100, Carl Zeiss, Germany). Optical biometry was performed with the IOLMaster 500 (Carl Zeiss Meditec, La Jolla, CA, USA) for intraocular lens (IOL) power calculation, and IOL power was determined using both the Barrett and SRK/T formulas. Preoperative evaluation included best-corrected visual acuity (BCVA, logMAR), intraocular pressure (IOP) measured using a fully automated non-contact tonometer (Canon, Japan), endothelial cell density (ECD) assessed with a specular microscope (CEM-530, NIDEK Co., Ltd., Gamagori, Japan), and central macular thickness (CMT) measured by optical coherence tomography (Cirrus HD-OCT Model 5000, Carl Zeiss Meditec, Dublin, CA, USA). The same parameters were reassessed postoperatively at six months. Cystoid macular edema (CME) was defined as the presence of intraretinal cystic changes on optical coherence tomography associated with increased central macular thickness. Changes in central macular thickness (CMT) were expressed as percentage change from preoperative values (ΔCMT%). Similarly, endothelial cell density (ECD) loss was calculated as percentage change from baseline (ΔECD%). Visual acuity change was defined as the difference between preoperative and postoperative logMAR values (ΔlogMAR).

In all cases with IOL subluxation, the Yamane technique was preoperatively planned prior to surgery. In contrast, in phacoemulsification cases, the decision to proceed with the Yamane technique was made intraoperatively in situations where capsular support was insufficient and sulcus IOL implantation was not feasible. Anterior vitrectomy was performed in all cases via a limbal approach in cases with vitreous prolapse into the anterior chamber. Pars plana vitrectomy was not performed in this series. In cases requiring cataract extraction, phacoemulsification and anterior vitrectomy were performed using the Alcon Centurion Vision System (Alcon Laboratories, Fort Worth, TX, USA).

The inclusion criteria were patients who underwent Yamane technique following IE or after phacoemulsification, with a minimum follow-up period of 6 months and complete clinical data. All consecutive eligible patients during the study period were included to minimize selection bias.

The exclusion criteria included patients with incomplete medical records, those who did not complete the required follow-up period, and patients younger than 18 years of age.

This study was approved by the Scientific Research Ethics Committee of Adana City Training and Research Hospital (Decision No. 1179) and adhered to the tenets of the Declaration of Helsinki. Written informed consent forms were obtained from all patients.

### Calculation of IOL-related astigmatism

IOL-related (internal) astigmatism following Yamane scleral fixation was calculated using vectorial analysis based on the Alpins method. Manifest refractive astigmatism and corneal astigmatism were converted into double-angle vector components, and the vectorial difference between these two parameters was defined as IOL-related astigmatism (ocular residual astigmatism). The magnitude and axis of the resulting vector were then reconverted into conventional cylinder notation. IOL tilt was assessed clinically based on slit-lamp biomicroscopy findings following pupillary dilation, as objective imaging modalities such as anterior segment OCT or ultrasound biomicroscopy were not routinely available.

### Surgical techniques

#### IE + Yamane Technique

**Fig. 1:**
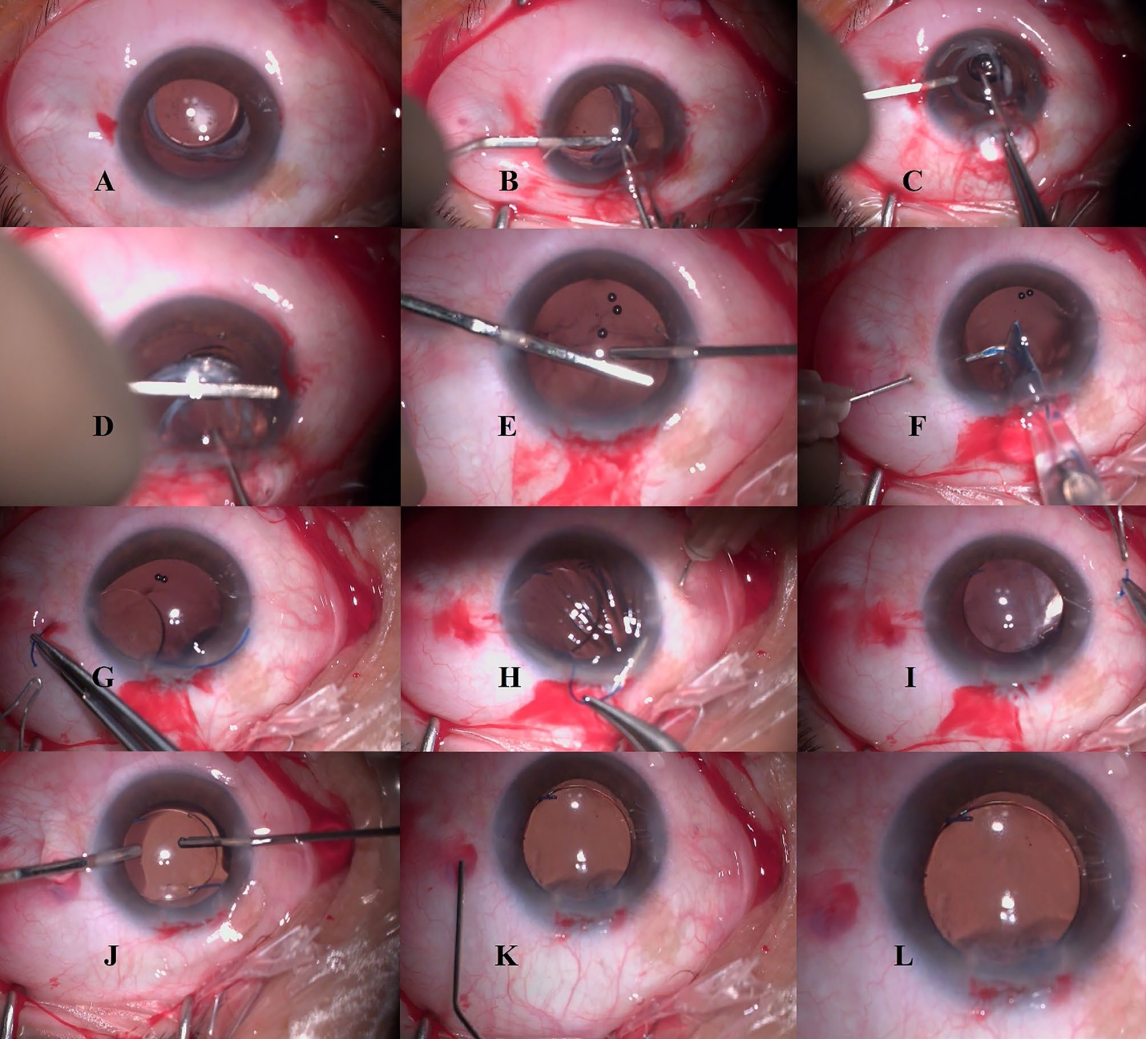
(**A**) subluxated IOL (**B**) repositioning of the subluxated IOL into the anterior chamber and externalization of one of the haptics through the main incision (**C**–**D**) IOL extraction using the “twist-and-out” technique with a spatula inserted through the side port as a protective barrier over the IOL to safeguard the corneal endothelium (**E**) anterior vitrectomy (**F**–**I**) sequential steps of the Yamane technique (**J**) repeat anterior vitrectomy with removal of residual viscoelastic material from behind the lens (**K**) burying of the cauterized haptics into the sclera (**L**) hydration of the side ports and completion of surgery without sutures or wound enlargement

In all cases, the periocular area was disinfected with 10% povidone-iodine and the surgical field was draped in a sterile fashion. The eyelashes and conjunctival sac were irrigated with 5% povidone-iodine, and a contact time of 3 minutes was allowed. Two side-port incisions were created using a microvitreoretinal (MVR) blade. Intracameral triamcinolone acetonide (Kenacort) was administered to assess the presence of vitreous in the anterior chamber. In cases where vitreous prolapse was detected, anterior vitrectomy was performed. In the absence of vitreous, the subluxated IOL was directly brought into the anterior chamber. After positioning the IOL in the anterior chamber, a viscoelastic substance was injected both above and below the IOL. In the IE group, all explanted IOLs were foldable single-piece IOLs. Cases involving refixation of three-piece IOLs or explantation of PMMA IOLs were not included in the study. The IOL was grasped using forceps and explanted through the main incision using the “twist-and-out” technique. During this maneuver, a spatula inserted through the side port was used as a protective barrier to prevent contact between the IOL and the corneal endothelium. Following IE, triamcinolone was re-administered and anterior vitrectomy was performed as needed. Subsequently, a 27-gauge insulin syringe needle was introduced transconjunctivally 2 mm posterior to the limbus, and the haptics were externalized sequentially through the sclera. The tips of the haptics were then cauterized to create flanged ends and securely embedded into the scleral tunnels. At the conclusion of the procedure, intracameral antibiotics were administered, and the surgery was completed without placement of corneal sutures. The Yamane technique was performed in the same surgical session in all cases. The surgical steps are presented in Fig. 1 as intraoperative photographs extracted from the corresponding surgical video. The complete procedure is further demonstrated in Supplementary Video 1.

#### Yamane technique after CCS

**Fig. 2:**
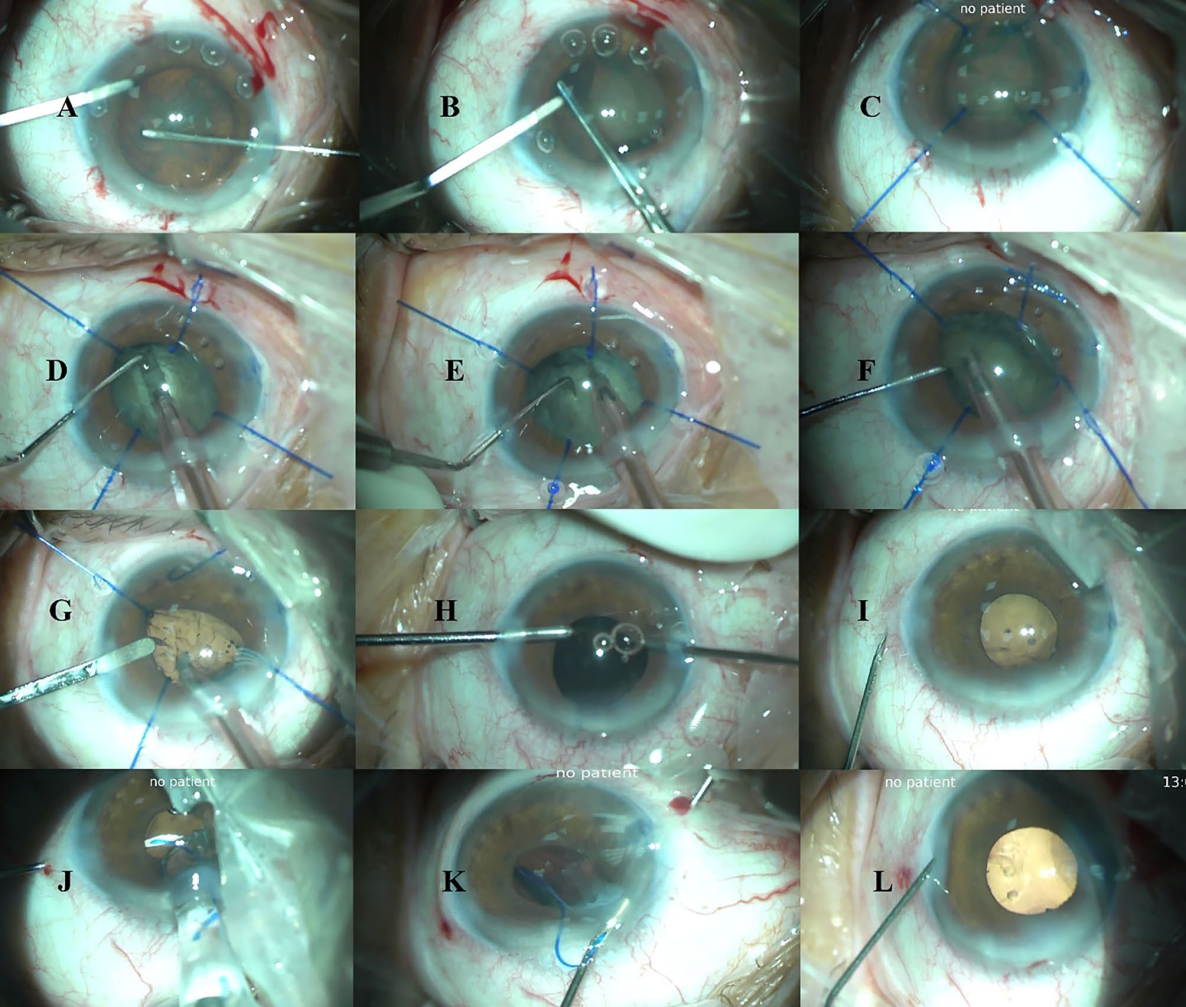
(**A**) difficulty during the initial capsulorhexis puncture, with marked capsular wrinkling, (**B**) temporal displacement of the capsular–lens complex during continuation of the capsulorhexis, (**C**) stabilization of the capsule using a capsular hook after completion of the capsulorhexis, (**D**) creation of the groove, (**E**) division of the nucleus into two equal halves, (**F**) phacoemulsification, (**G**) at the end of phacoemulsification, the posterior capsule remained intact; however, marked zonular weakness was observed inferonasally following disengagement of the capsular hook, (**H**) capsular cleanup with anterior vitrectomy, (**I**–**K**) sequential steps of the Yamane technique, (**L**) hydration of the side ports and completion of surgery without sutures or wound enlargement

In all cases, the periocular area was disinfected with 10% povidone-iodine and the surgical field was draped in a sterile fashion. The eyelashes and conjunctival sac were irrigated with 5% povidone-iodine, and a contact time of 3 minutes was allowed. Two side-port incisions were created using a microvitreoretinal (MVR) blade. After injection of viscoelastic material into the anterior chamber, a 2.75-mm main corneal incision was created. Continuous curvilinear capsulorhexis was then performed. In cases with significant phacodonesis, capsular hooks were applied to provide adequate capsular support, followed by phacoemulsification of the nucleus. After removal of the epinucleus and cortical material, the Yamane technique was planned in eyes with insufficient capsular support or complete capsular bag dislocation. In these cases, the capsular bag was removed using capsular forceps to prevent postoperative capsular opacification. Intracameral triamcinolone acetonide (Kenacort) was then administered, and anterior vitrectomy was performed. Subsequently, a 27-gauge insulin syringe needle was introduced transconjunctivally 2 mm posterior to the limbus, and the haptics were externalized sequentially through the sclera. The tips of the haptics were cauterized to create flanged ends and then securely embedded into the scleral tunnels. At the conclusion of the procedure, intracameral antibiotics were administered, and the surgery was completed without placement of corneal sutures. The Yamane technique was performed in the same surgical session in all cases. The surgical steps are presented in Figure 2 as intraoperative photographs extracted from the corresponding surgical video. The complete procedure is further demonstrated in Supplementary Video 2.

### Statistical analysis

Statistical analyses were performed using IBM SPSS Statistics (version 26.0; IBM Corp., Armonk, NY, USA). Normality of continuous variables was assessed using the Shapiro–Wilk test. Data were presented as mean ± standard deviation or median (interquartile range), depending on distribution. Within-group preoperative and postoperative comparisons were conducted using the paired-samples t test for normally distributed variables and the Wilcoxon signed-rank test for non-normally distributed variables. Between-group comparisons of continuous variables were performed using the Mann–Whitney U test. Categorical variables are presented as frequencies and percentages and were compared using Fisher’s exact test. A post-hoc power analysis was performed for the primary outcome (ΔBCVA) using G*Power software (Heinrich Heine University, Düsseldorf, Germany). All statistical analyses were two-tailed, and a *p* value < 0.05 was considered statistically significant.

## Results

A total of 50 patients were included in the study, of whom 19 (38%) were female and 31 (62%) were male. Thirteen patients were included in the CCS group, while 37 patients were included in the IE group. In the CCS group, 7 patients were female and 6 were male, whereas the IE group consisted of 12 female and 25 male patients. There was no statistically significant difference in gender distribution between the groups (*p* = 0.199). There was no statistically significant difference in age between the groups (*p* = 0.825). The median follow-up time was 7 [6–8] months in the CCS group and 7 [6–8] months in the IE group.

When the etiologies were reviewed, among patients with IOL subluxation, a history of trauma was present in 12 of 37 patients, pseudoexfoliation (PEX) in 15 patients, and iatrogenic zonular weakness secondary to previous complicated cataract surgery in 10 patients. In the CCS group (*n* = 13), PEX was present in 10 patients, while 3 patients had a history of trauma.

Analysis of spherical equivalent values revealed a significant postoperative improvement in the CCS group (*p* < 0.001). Similarly, the IE group also demonstrated a significant change in spherical equivalent from preoperative values (*p* < 0.001). In the CCS group, absolute astigmatism decreased postoperatively; however, this reduction did not reach statistical significance (*p* = 0.124). In contrast, the IE group demonstrated a significant postoperative decrease in absolute astigmatism (*p* = 0.036). IOL-related astigmatism was low and comparable between groups, with no statistically significant difference (*p* = 0.765). IOL tilt was observed in two patients (4%), both in the IE group, and was associated with increased postoperative astigmatism. Despite these relatively high values, neither patient required revision surgery, as satisfactory visual acuity was achieved with spectacle correction.

BCVA improved significantly in both groups, with no statistically significant difference in the magnitude of improvement between groups (ΔlogMAR, *p* = 0.063).

Intraocular pressure (IOP) outcomes differed between the groups. In the CCS group (*n* = 13), IOP decreased significantly after surgery (*p* = 0.036). In contrast, the IE group (*n* = 37) showed no significant difference between preoperative and postoperative IOP values (*p* = 0.476).

Endothelial cell density (ECD) decreased significantly after surgery in both groups (*p* = 0.046 and *p* < 0.001, respectively). Percentage endothelial cell loss (%ECD) was calculated for standardized comparison, and considerable variability was observed in both groups, including occasional negative values, likely reflecting measurement variability rather than true biological increase. When expressed as percentage change, %ECD loss was comparable between the groups, with no statistically significant difference (*p* = 0.382).

Central macular thickness remained stable after secondary intraocular lens implantation using the Yamane technique in both surgical groups, with no statistically significant postoperative change observed in within-group analyses. Overall, surgery was not associated with significant macular thickening in either group. Cystoid macular edema (CME) was identified in 3 (6%) cases: one in the CCS group and two in the IE group. All affected eyes were treated with a sub-Tenon triamcinolone (Kenacort) injection, leading to complete resolution of macular edema. There was no statistically significant difference in the percentage change in central macular thickness between the CCS group and the IE group.

When postoperative complications were evaluated, subgroup analysis showed that cystoid macular edema (CME) occurred in 1 of 13 eyes (7.7%) in the CCS group and in 2 of 37 eyes (5.4%) in the IE group. Subconjunctival hemorrhage was observed in 5 of 13 eyes in the CCS group and in 21 of 37 eyes in the IE group. No postoperative intraocular pressure elevation requiring medical treatment was observed in any case. In the IE group, IOL tilt secondary to haptic memory loss was observed in 2 cases. No cases of hyphema, vitreous hemorrhage, or retinal detachment were observed in either group.

The within-group analyses for the CCS and IE groups, as well as the between-group comparisons, are presented in Tables 1 and 2, respectively.

**Table 1:** Within-group analysis of CCS and IE groups

Parameter	CCS Preoperative	CCS Postoperative	p-value	IE Preoperative	IE Postoperative	p-value
Spherical Equivalent (D)	−2.25 ± 7.08	0.07 ± 1.17	<0.001	11.25 (7.38)	−0.50 (1.44)	<0.001
Astigmatism (D)	−3.25 (1.50)	−1.75 (1.38)	0.124	−2.75 (2.13)	−1.75 (1.75)	0.036
BCVA (logMAR)	1.52 (0.50)	0.30 (0.30)	0.001	1.30 (0.40)	0.30 (0.39)	<0.001
IOP (mmHg)	17.23 ± 6.95	14.54 ± 5.97	0.036	13 (6.0)	14 (6.50)	0.476
ECD (cells/mm^2^)	2495 (711)	2258 (1174.5)	0.046	2099 (661.50)	2023 (1168.50)	<0.001
CMT (µm)	259 (46.5)	260 (33)	0.576	258 (60.50)	262 (52.00)	0.322

(normally distributed variables are presented as mean ± SD, and non-normally distributed variables are presented as median (IQR), based on Shapiro–Wilk test results)

**Table 2:** Between-group comparison (CCS vs IE)

Parameter	CCS Group	IE Group	p-value
Age (years, median, IQR)	74 (10)	73 (13)	0.825
Gender (female/male)	7/6	12/25	0.199
Follow-up time (months, median, IQR)	7 (2)	7 (2)	0.230
ΔBCVA (logMAR) (mean ± SD)	1.25 ± 0.61	0.94 ± 0.47	0.063
% ECD Loss (median, IQR)	5.5% (27.2)	9.0% (32.5)	0.382
% CMT Increase (median, IQR)	0.39% (13.77)	2.18% (10.19)	0.974
IOL-related astigmatism (median, IQR)	−0.79 (0.73)	−0.71 (0.69)	0.765

(normally distributed variables are presented as mean ± SD, and non-normally distributed variables are presented as median (IQR), based on Shapiro–Wilk test results)

## Discussion

Phacodonesis is defined as abnormal mobility of the crystalline lens due to zonular weakness or dehiscence, whereas IOL subluxation refers to partial displacement of an implanted lens caused by insufficient capsular or zonular support. Both conditions are associated with zonular instability and share common risk factors, including advanced age, pseudoexfoliation syndrome, high myopia, ocular trauma, prior intraocular surgery, chronic uveitis, and systemic connective tissue disorders such as Marfan syndrome and homocystinuria. In addition, long-term corticosteroid use and previous vitreoretinal surgery may further impair zonular integrity, increasing the risk of lens instability [15–17]. In the present study, the distribution of predisposing factors was consistent with the literature, with pseudoexfoliation (PEX) and ocular trauma emerging as the most common etiologies in both groups. These findings align with previous reports identifying PEX-related zonular weakness and trauma-induced mechanical disruption as major contributors to lens instability. Additionally, iatrogenic zonular damage following prior complicated cataract surgery was prominent in the IE group, underscoring the impact of surgical factors on capsular support. The predominance of PEX, particularly in the CCS group, further supports its well-established association with progressive zonular degeneration. Overall, our results confirm that lens instability is multifactorial and primarily linked to conditions affecting zonular integrity. In such cases, the management of aphakia following phacoemulsification or IOL extraction represents a significant clinical challenge. As demonstrated in multiple studies in the literature, Yamane fixation is a well-established surgical technique for the management of aphakia, providing significant improvements in visual outcomes [18–22]. However, in pseudophakic eyes with subluxated IOLs, the presence of an existing lens makes IOL-preserving approaches particularly attractive. In this context, scleral fixation of the patient’s existing IOL has been described in the literature as a viable option, allowing stabilization of the lens while avoiding explantation-related complications and eliminating the need for sutures, knots, or glue [23]. Nevertheless, the technique requires meticulous surgical execution to achieve symmetric haptic tension, as imbalance may result in IOL tilt or decentration, and the long-term implications of subtenon flange positioning remain uncertain. In a comparative study by Icoz et al., both modified Yamane and double-flange techniques showed similar efficacy and safety; however, Yamane fixation provided superior postoperative stability, with lower cylindrical error, reduced pseudophacodonesis, and decreased central macular thickness, suggesting better IOL centration and fewer micro-movements [21]. In our study, it was preferred primarily based on evidence from the literature supporting its superior stability, and secondarily due to the surgeon’s greater familiarity with the procedure, particularly in cases requiring IOL exchange.

Previous studies have specifically investigated the optimal timing of the Yamane technique in cases with insufficient capsular support. In a recent study by Icoz et al., no significant differences were observed between same-session and second-session applications in terms of postoperative visual acuity, refractive outcomes, or intraocular pressure. However, early postoperative complications, such as cystoid macular edema, corneal edema, and transient intraocular pressure elevation, were reported more frequently in the same-session group, although no differences were detected in late postoperative complications [22]. In our study, all cases were managed in a single surgical session. This approach was preferred to avoid the need for a second surgical intervention, reduce additional intraocular manipulation, and provide earlier visual rehabilitation. Furthermore, single-session surgery may offer practical advantages by minimizing repeated hospital visits and reducing the overall treatment burden for both patients and the healthcare system.

Previous studies have consistently demonstrated significant improvements in visual acuity following Yamane fixation [24–26]. In our study, in line with the existing literature, both surgical approaches resulted in statistically and clinically significant gains in visual acuity. Best-corrected visual acuity improved markedly after Yamane fixation regardless of whether the procedure was preceded by complicated cataract surgery or intraocular lens explantation, underscoring the effectiveness of the Yamane technique in managing aphakia following either surgical intervention.

One of the most challenging steps in post-CCS and post-IE management is secondary IOL implantation, as it is essential for achieving a refractive target close to emmetropia. In the literature, several studies have demonstrated that the Yamane technique for sutureless intrascleral IOL fixation is effective in approaching emmetropia in terms of spherical equivalent outcomes [27, 28]. In line with these reports, our results also indicate that Yamane-based IOL implantation can reliably bring the refractive status closer to emmetropia after both CCS and IE, supporting the technique’s effectiveness in achieving satisfactory postoperative refractive outcomes. Although no statistically significant difference was observed between the groups, the apparent difference in the magnitude of change may have a plausible explanation, particularly considering the removal of cataractous lenses in the CCS group. Moreover, the *p*-value approaching the threshold of significance and the post-hoc power of approximately 62% suggest that the study may have been underpowered to detect moderate between-group differences; therefore, larger prospective studies are warranted. One of the main challenges in cases with IOL or lens subluxation due to zonular weakness is the presence of high preoperative astigmatism. Since the capsular bag cannot be preserved in these eyes, achieving stable fixation with scleral techniques is crucial, and one of the primary goals is to reduce the high preoperative astigmatism and obtain low postoperative astigmatism. Indeed, studies in the literature have demonstrated the success of the Yamane technique in this regard [26, 29, 30]. In our study, postoperative astigmatism decreased in both groups, and this reduction reached statistical significance in the IE group. In the CCS group, although a numerical reduction in postoperative astigmatism was observed, statistical significance was not achieved. This discrepancy, despite a magnitude of reduction comparable to that observed in the IE group, is most likely attributable to a type II error related to the small sample size in the CCS group (*n* = 13). The limited number of patients in this subgroup reduces the statistical power to detect moderate differences. Therefore, the absence of statistical significance should not be interpreted as evidence of lack of effect, but rather as a potential consequence of insufficient sample size. Larger prospective studies with adequately powered sample sizes are required to more definitively evaluate these differences.

Notably, the median IOL-related astigmatism in our series remained below 1.0 D in both groups, with values of −0.79 D (IQR: 0.73) in the CCS group and −0.71 D (IQR: 0.69) in the IE group, indicating a low and clinically acceptable level of internal astigmatism following Yamane scleral fixation. In comparison, İcöz et al. reported higher postoperative lenticular astigmatism values of approximately −1.04 ± 0.26 D at 1 month and −1.14 ± 0.32 D at 6 months following a modified double-flanged technique, despite maintaining good IOL stability without tilt or decentration [14]. The lower internal astigmatism in our study may suggest that the Yamane technique provides more stable IOL positioning, potentially resulting in reduced optical aberrations and improved refractive outcomes. This is further supported by a comparative study by İcöz et al., in which the modified Yamane technique demonstrated significantly lower cylindrical refractive error and reduced pseudophacodonesis compared to the modified double-flanged technique, suggesting improved IOL stability and centration [21]. This favorable astigmatic profile may also be partly attributed to the use of the Eyecryl intraocular lens, which appears well-suited for the Yamane technique in terms of haptic behavior and structural properties. This interpretation is supported by previous studies demonstrating that, although different IOL models yield comparable levels of postoperative astigmatism, variations in haptic design, rigidity, and shape memory can influence IOL centration, tilt, and overall refractive predictability [31, 32]. Therefore, variability in astigmatic outcomes across different studies may, at least in part, reflect differences in IOL material and haptic characteristics, as well as the surgeon’s familiarity with the specific biomechanical properties of the implanted lens. In this context, both appropriate IOL selection and surgeon experience appear to play a critical role in achieving predictable and clinically acceptable outcomes following Yamane scleral fixation.

Enlargement of the main incision during IE is a well-recognized factor contributing to increased postoperative astigmatism, particularly in secondary IOL surgery. In the present study, in cases requiring IE, the subluxated lenses were removed in a single piece using the twist-and-out technique described by Pandit et al. [33]. This methodological approach likely played a key role in preserving corneal integrity and minimizing surgically induced astigmatism. Therefore, the relatively stable postoperative astigmatism observed in our series may, at least in part, be attributed to the consistent use of this technique. The risk of IOL tilt, which is also known to occur with other scleral fixation methods, has been reported in the literature [18, 34–36]. In line with these reports, two cases in our series (4%) demonstrated marked postoperative astigmatism associated with IOL tilt, both occurring in the IE group. Examinations performed after pupillary dilation revealed loss of haptic memory and bending in these eyes. Importantly, in both cases, tilt was associated with the second haptic, where surgical manipulation during externalization was more challenging, potentially leading to suboptimal positioning and increased mechanical stress on the haptic. In addition, improper needle entry angle or asymmetrical scleral fixation may also contribute to the development of IOL tilt. These findings highlight the importance of precise needle angulation and symmetric haptic externalization during the Yamane technique to ensure optimal IOL centration and stability. Despite the presence of IOL tilt, both patients achieved satisfactory visual outcomes with spectacle correction and declined revision surgery.

In the present study, IOP outcomes differed between the surgical subgroups. None of the patients in either group had a preexisting diagnosis of glaucoma, and no patient required antiglaucomatous medication during postoperative follow-up; therefore, glaucoma was not considered a confounding factor in the intraocular pressure analysis. In the CCS group, IOP decreased significantly after surgery. This IOP reduction aligns with findings from Zamani et al. [37], who reported significant decreases in IOP at 1 and 6 weeks after uncomplicated phacoemulsification, and Todorović et al. [38], who observed early postoperative IOP reductions in healthy phakic eyes. By contrast, in the IE group, no significant postoperative change in IOP was observed. This finding is consistent with the results of Anggara et al. [39], who reported stable preoperative and 1-month postoperative IOP in eyes undergoing Yamane scleral fixation, evaluating both phakic and aphakic eyes together. The lack of significant IOP change in these eyes likely reflects the fact that the IE subgroup had already undergone prior lens removal, so the main IOP-lowering effect of lens extraction had already occurred. Taken together, these findings indicate that significant postoperative IOP reduction is primarily observed in phakic eyes undergoing lens extraction, whereas eyes undergoing secondary Yamane fixation after prior IE tend to maintain stable intraocular pressure, in line with the existing literature.

In the present study, ECD decreased significantly after surgery in both the CCS and IE groups. The observed within-group reductions in ECD confirm that endothelial stress remains an inherent concern in these complex surgical settings. When contextualized within the existing literature, the magnitude of endothelial cell loss observed in the present study appears comparable to previously reported Yamane series. In the original case series by Yamane et al. [40], mean endothelial cell loss at 3 months was approximately 6.0 ± 7.3%, which closely aligns with the median percentage ECD loss observed in both groups in our study. Similarly, Saeki et al. [34] reported a postoperative decrease in ECD from 2282 to 2091 cells/mm^2^, corresponding to an endothelial cell loss of approximately 8.4%, again comparable to our findings. In a prospective comparative study, Błagun et al. [41] documented a mean ECD loss of 3.5% in the Yamane group. Taken together, these results indicate that the degree of endothelial cell loss observed in our cohort falls within the range reported in the literature for Yamane fixation.

Despite statistically significant within-group ECD reductions, standardized comparison using percentage endothelial cell loss revealed no statistically significant difference between the phakic and IE groups. However, the IE group showed a numerically higher median percentage of ECD loss than the CCS group (9.0% vs. 5.5%). This apparent difference may be explained by the lower baseline endothelial cell reserve in the IE group. Although the absolute number of endothelial cells lost was likely similar between the groups, the reduced baseline reserve in the IE group would result in a higher percentage loss. In cases with reduced endothelial reserve, additional precautions should be undertaken to minimize endothelial trauma. Accordingly, in our series, during the twist-and-out maneuver, a spatula was introduced through the side port to serve as a mechanical barrier, preventing contact between the IOL and the corneal endothelium during extraction. In addition, a dispersive viscoelastic was applied over the anterior surface of the IOL to provide further endothelial protection and reduce the risk of mechanical injury. From a clinical perspective, particular emphasis should be placed on meticulous surgical technique and enhanced intraoperative precautions in eyes with a lower preoperative endothelial reserve, especially in cases undergoing secondary procedures.

In the present study, central macular thickness remained stable following secondary intraocular lens implantation with the Yamane technique, indicating preservation of macular architecture regardless of the preceding surgical approach. No significant macular thickening was observed in either the CCS-preceded Yamane group or the Yamane fixation following IE group. Postoperative cystoid macular edema was observed in 6% of cases overall, occurring in 7.7% of eyes in the CCS group and 5.4% in the IE group. All cases were successfully treated with sub-Tenon triamcinolone (Kenacort) injection, resulting in complete resolution. Reported CME rates following Yamane fixation in the literature vary from 2.5% to 14%, with Yalçınbayır et al. [42] reporting 2.5%, Vural et al. [43] reporting 3.3%, Ishikawa et al. [44] reporting 13%, and Icoz et al. [14] reporting 14%. The overall and subgroup CME incidence observed in our study falls within this reported range, supporting that secondary IOL implantation using the Yamane technique is associated with both stable macular thickness and a low incidence of CME, consistent with previous findings. However, it should not be forgotten that there is still a risk of postoperative macular edema, and routine OCT follow‑ups are recommended.

Complications associated with the Yamane technique reported in the literature include cystoid macular edema, IOL tilt or decentration, IOL drop into the vitreous cavity, haptic-related complications such as deformation, breakage, or disinsertion at the haptic–optic junction, iris-related complications including iritis, iridodialysis, and uveitis–glaucoma–hyphema (UGH) syndrome, as well as subconjunctival hemorrhage, intraocular pressure elevation, vitreous hemorrhage, suprachoroidal hemorrhage, and retinal detachment [45–47]. In our study, the observed complications were limited to cystoid macular edema, IOL tilt, and subconjunctival hemorrhage. All cases of cystoid macular edema were successfully managed with medical treatment, while cases with IOL tilt were managed conservatively with spectacle correction based on patient preference. Subconjunctival hemorrhage was relatively frequent in both groups, particularly in the IE group; however, this finding is generally benign and self-limiting, reflecting scleral manipulation rather than a clinically significant adverse event. Importantly, no cases of postoperative intraocular pressure elevation requiring treatment were observed, supporting previous evidence that Yamane fixation is associated with a low risk of sustained IOP elevation. Finally, the absence of severe complications in both groups further supports the overall safety of the Yamane technique. Taken together, these findings indicate that Yamane fixation, whether performed following CCS or IE, provides a reliable and safe surgical option, with complication profiles largely reflecting known mechanisms related to IOL manipulation, fixation stability, and intraocular inflammation.

This study has several limitations that should be acknowledged. Although a total of 50 eyes were included, the distribution between groups was unbalanced, with only 13 eyes in the CCS group, which may have reduced statistical power. This imbalance, particularly the relatively small sample size of the CCS group, should be considered when interpreting between-group comparisons, as it may limit the ability to detect clinically relevant differences. Consequently, some comparisons may have been underpowered, and the absence of statistical significance in certain parameters may reflect a type II error rather than the absence of a true clinical effect. In line with this, post-hoc power analysis for ΔBCVA indicated a statistical power of approximately 62%, further supporting the possibility of insufficient power to detect moderate between-group differences. Another important limitation is the lack of objective imaging modalities, such as anterior segment optical coherence tomography or ultrasound biomicroscopy, to directly assess IOL centration and tilt. However, IOL-related astigmatism was evaluated using vectorial analysis based on the Alpins method, which provides an indirect but functionally relevant assessment of IOL positioning and stability. In addition, the retrospective design may introduce selection and information bias, and the single-center setting may limit the generalizability of the findings. Furthermore, all surgeries were performed by a single surgeon, which may introduce operator-dependent bias and limit the external validity of the results. Another consideration relates to the variability inherent in specular microscopy measurements. Previous studies have reported coefficients of variation of approximately 3–4% under ideal conditions, which may increase to 5–10% in routine clinical practice [48]. In this context, the occasional negative values observed in percentage endothelial cell density (ECD) loss in our study are most likely attributable to measurement variability rather than true biological increase. Notably, these values were limited in number, predominantly observed in a small subset of patients in the IOL extraction group, and remained below the variability range reported in the literature. These findings were more apparent in cases with minimal surgical trauma and well-preserved endothelial integrity. Therefore, to preserve real-world data variability and avoid introducing bias, these cases were not excluded from the analysis; however, their interpretation has been clearly acknowledged. Finally, the follow-up period was limited to six months; therefore, the present findings should be interpreted as short-term outcomes, and longer-term studies are needed to confirm the durability and safety of these results.

## Conclusion

Secondary intraocular lens implantation using the Yamane technique provides effective visual and refractive rehabilitation with stable anatomical outcomes, regardless of prior surgical approach. Both CCS- and IE-preceded cases achieved comparable results, with predictable refractive outcomes and preserved macular and corneal integrity, demonstrating consistent performance across different clinical scenarios. While endothelial cell loss is an inherent consideration, our results demonstrate a similar magnitude of loss between the groups, underscoring the importance of endothelial-protective strategies. Intraocular pressure behavior differed according to prior lens status, with reduction observed in phakic eyes and stability in previously operated eyes, reflecting expected physiological responses. Overall, the low complication profile and consistent functional outcomes support the Yamane technique as a safe, reliable, and versatile option for secondary IOL implantation.

## Electronic supplementary material

Below is the link to the electronic supplementary material.

Supplementary Material 1

Supplementary Material 2

## Data Availability

The data analyzed during the current study are not publicly available due to ethical restrictions and the protection of personal data in accordance with applicable data protection regulations. However, the datasets are available from the corresponding author upon reasonable request and subject to appropriate ethical approval.
